# Development and Validation of a New UFLC–MS/MS Method for the Detection of Organophosphate Pesticide Metabolites in Urine

**DOI:** 10.3390/molecules28155800

**Published:** 2023-08-01

**Authors:** Dileshwar Kumar, Sukesh Narayan Sinha, Kasturi Vasudev

**Affiliations:** 1 Food Safety Division, ICMR—National Institute of Nutrition, Hyderabad 500007, Telangana, India; 2 Department of Biochemistry, Osmania University, Hyderabad 500007, Telangana, India

**Keywords:** biomarker, DAP metabolites, organophosphate pesticides, UFLC–MS/MS, urine

## Abstract

To monitor human exposure to pesticides, experts commonly measure their metabolites in urine, particularly dialkyl phosphates (DAPs), which include diethyl phosphate (DEP), Diethyl thiophosphate (DETP), diethyl dithiophosphate (DEDTP), dimethyl phosphate (DMP), dimethyl thiophosphate (DMTP) and dimethyl dithiophosphate (DMDTP)to monitor the metabolites of organophosphates. These DAP metabolites are a urinary biomarker for assessing pesticide exposure and potential health risks. This study presented a new screening method combining ultrafast liquid chromatography with tandem mass spectrometry (UFLC–MS/MS) to detect six DAP metabolites in human urine. The study also compared standard sample extraction methods, namely, liquid–liquid extraction (LLE); quick, easy, cheap, effective, ruggedand safe (QuEChERS); and lyophilization. After a comprehensive analysis of the methods used to extract the analytes, including recovery rate, repeatability and reproducibility, the liquid–liquid extraction (LLE) method was found to be the best. It had a high recovery rate, was easy to handle, required less sample volume and had a short extraction time. Therefore, the LLE method was chosen for further analysis. The results showed excellent performance with high recoveries between 93% and 102%, precise repeatability (RSD) between 0.62% and 5.46% and acceptable reproducibility values (RSD) between 0.80% and 11.33%. The method also had limits of detection (LOD) ranging from 0.0201 ng/mL to 0.0697 ng/mL and limits of quantification (LOQ) ranging from 0.0609 ng/mL to 0.2112 ng/mL. Furthermore, the UFLC–MS/MS method was validated based on the SANTE guidance and successfully analyzed 150 urine samples from farmers and non-farmers. This validated method proved useful for biomonitoring studies focusing on OP pesticide exposure. It offers several advantages, such as a reduced need for samples, chemicals and materials, and a shorter analysis time. The method is sensitive and selective in detecting metabolites in human urine, making it a valuable approach for the practical and efficient assessment of pesticide exposure.

## 1. Introduction

Organophosphate pesticides (OP) are toxic substances that eliminate pests in households and on agricultural land [1]. However, their negative impact on the environment and human health is a global problem [2], especially in developing countries [3,4]. Pesticide poisoning depends on the duration, frequency and amount of exposure. acute and chronic exposure to pesticides has adverse health effects and can occur during and after exposure [5]. The OP insecticides are neurotoxins that can cause permanent or reversible damage to the structure or function of the nervous system [6], primarily affecting the neurotransmitter acetylcholine by inhibiting the activity of the enzyme acetylcholinesterase in humans [7]. There are many routes of exposure to pesticides, leading to human exposure through various pathways [8].

In recent epidemiological studies, various biomarkers have been used to measure the impact of pesticide exposure on health. Over the past two decades, researchers have explored the potential health biomarkers of pesticides and their metabolites in biological samples such as serum, lipids, urine, blood and breast milk. These biomarkers include acetylcholinesterase (AChE), an enzymatic marker [9], oxidative markers such as malondialdehyde (MDA) and reduced glutathione (GSH), and DNA damage markers such as micronuclei [10,11]. In addition, there is still a need for comprehensive assessments of the use of DAP metabolites as biomarkers of organophosphate pesticide poisoning. Several studies have shown that higher concentrations of DAP metabolites are associated with various neurological and other health markers. These biomarkers are useful to estimate overall exposure; a recent study found that exposure to low doses of OP was associated with several adverse health effects [12], including neurological disorders [13,14], and changes in hormone profiles [15]. The toxic effects of pesticide exposure have been observed in rural, urban and workplace settings [16,17]. Studies have also shown that exposure to pesticides is associated with non-communicable diseases [18], and exposure during pregnancy has been linked to higher concentrations of pesticide metabolites. In addition, higher DAP concentrations in the second half of pregnancy were found to be more strongly associated with IQ at age seven [19]. Higher DAP concentrations have also been associated with endocrine disruption in males and females [20,21,22], and asthma [23].

The OP pesticides have a short half-life, lasting only a few hours to a few days, and rapidly convert into pesticide-specific metabolites [24].These metabolites, known as dialkyl phosphate (DAP) [25], are converted into six common DAP metabolites when ingested: diethyl phosphate (DEP), diethyl thiophosphate (DETP), diethyl dithiophosphate (DEDTP),dimethyl phosphate (DMP), dimethyl thiophosphate (DMTP) and dimethyl dithiophosphate (DMDTP) [26,27]. The chemical structure of six urinary metabolites has given in Figure S1 (Supplementary). Therefore, the detection of these metabolites in urine within 24–48h indicates recent exposure. OP pesticides can also be degraded in the environment, metabolized by plants or degraded during food processing, leading to the presence of these metabolites in food and the environment [28]. The measurement of DAP metabolites in urine facilitates the monitoring of acute or occupational exposure to pesticides and the assessment of the potential health effects of such exposure on individuals.

Appropriate analytical methods are required to monitor exposure to organophosphate pesticides as these pesticides are widely used in agriculture, households and gardens worldwide, including in India [4]. Monitoring of DAP metabolites is crucial as they serve as potential biomarkers of population exposure to OP pesticides and hence sensitive and selective analytical methods are required. In the past, several analytical methods have been described for the measurement of DAP metabolites in urine and other matrices, including gas chromatography (GC) and liquid chromatography (LC) in combination with different detectors such as flame photometric detector (FPD) [29], nitrogen phosphorus detector (NPD) [30] and mass spectrometry (MS) [27,31,32,33,34,35,36]. Currently, LC–MS/MS is the main analytical method for monitoring pesticides and their metabolites in urine, with numerous methods published in recent years. These methods follow a specific analytical approach that involves the development of targeted methods focusing on a limited number of analytes (usually 2–6) and the application of sample treatments such as dilution, liquid–liquid Extraction(LLE), QuEChERS, Solid-Phase Extraction (SPE) and lyophilization [37,38,39]. However, the ability to analyze compounds with different physicochemical properties is simultaneously limited. To overcome this, need to develop new comprehensive quantitative analytical protocols for the six potentially most important biomarkers of exposure to multiple organophosphate pesticides to effectively measure the DAP metabolites and use them for human health risk assessment.

The aim of this study was to develop a reliable and sensitive method for detecting trace amounts of DAP metabolites in urine using UFLC–MS/MS analysis which is cost-effective and practical that any laboratory can use. We achieved this by implementing a simple extraction procedure and validating the method in terms of linearity, recovery, precision, accuracy, matrix effect and other relevant parameters. This innovative approach combines quantitative target analysis with improved analytical capabilities and enables the simultaneous analysis of multiple compounds in a single run. This method is a significant advancement in the field. It provides researchers and laboratories with a powerful tool to accurately determine the concentrations of DAP metabolites in urine and to study the effects of pesticide exposure on health and the environment.

## 2. Results and Discussion

### 2.1. Sample Preparation

To obtain accurate results, it is important to use an extraction method that maximizes analyte recovery and minimizes matrix effects. In this study, we evaluated the efficiency of three extraction methods—QuEChERS, lyophilization and LLE—for the measurement of metabolites. Our aim was to determine the most effective method that reduces endogenous interference. After a thorough evaluation, we found that the LLE method was particularly efficient in the analysis of DAP, a specific metabolite of interest. This method showed higher recoveries and lower matrix effects compared to QuEChERS and lyophilization. Therefore, we chose the LLE method as the preferred extraction method for the analysis of DAP in this study. In the LLE method, 200 μL of the urine sample and 100 μL of the standard are added to a 2 mL Eppendorf tube, followed by the addition of 800 μL of cold ethyl acetate. The mixture is shaken for 1 min and then placed on ice for 10 min to precipitate. The mixture is then centrifuged at 10,000 rpm for 10 min and the resulting supernatant is transferred to a 10 mL tube. The product is dried under nitrogen, reconstituted with 500 μL acetonitrile (ACN) and finally transferred to a vial for analysis UFLC–MS/MS. The samples preparation on comparision of different samples extraction procedure shown in Figure S4 (Supplementary).

### 2.2. Method Validation

#### 2.2.1. Selectivity

**Figure 1:**
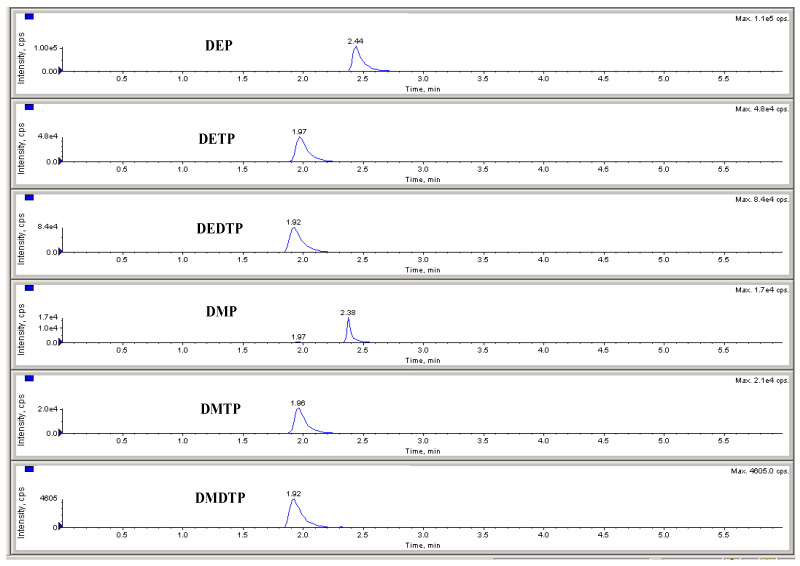
Chromatogram of DAP metabolites at a concentration of 100 ng/mL.

This method is highly selective because the transition from parents to daughters is specific for all analytes and the separation of all analytes occurs at different retention times, as shown in Figure 1. To test the selectivity of this method, six urine samples from healthy individuals were combined into a mixture spiked with the standard DAP. The blank and spiked urine samples were then analyzed using UFLC–MS/MS and it was found that no other interfering peaks occurred during chromatographic separation, indicating the selectivity of this method in detecting six DAP metabolites in urine samples.

#### 2.2.2. Linearity

The linearity was assessed by choking the 10-point calibrate adjustedfor the six DAP metabolites. Linear regression quantification was performed over a concentration range of 0.1 to 200 ng/mL (Table 1), with calibration points at 0.1, 0.5, 1, 5, 10, 25, 50, 100, 150 and 200 ng/mL. The regression analysis yielded the following equations: DEP Y = 9729.7x−10,842 (R^2^ = 0.9997), DETP Y = 8704.9x + 13,258 (R^2^ = 0.999), DEDTP Y = 19,603x + 123,119 (R^2^ = 0.9964), DMP y = 19,603x + 123,119 (R^2^ = 0.9964), DMTP y = 2196x + 6261.6 (R^2^ = 0.998) and DMDTP y = 2196x + 6261.6 (R^2^ = 0.9983). The linearity graph of six metabolites have shown in Figure S2 (Supplementary).

#### 2.2.3. LOQ and LOQ

The measurement of the lower limit of detection and quantification of this analytical method were carried out using standard analytes at concentrations of 0.1–200 ng/mL. For the detection and measurement of DAP metabolites in human urine with UFLC–MS/MS, a signal-to-noise ratio of 3:1 for LOD and 10:1 for LOQ was used. This method can identify low concentrations of, DEP, DETP, DEDTP, DMP, DMTP and DMDTP with a detection limit (LOD) of 0.0201, 0.0323, 0.0697, 0.0207, 0.0488 and 0.0406 ng/mL, respectively. The lower limit of quantification for, DEP, DETP, DEDTP, DMP, DMTP and DMDTP is 0.0609, 0.0969, 0.2112, 0.0626, 0.1479 and 1.229 ng/mL, respectively (Table 2).

#### 2.2.4. Recovery

**Figure 2:**
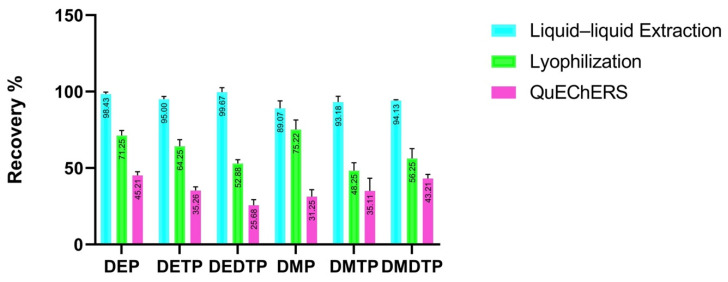
Comparison of extraction recovery of three extraction methods in three concentration ranges.

The recovery was tested with three different extraction procedures: lyophilization, QuEChERS and liquid–liquid extraction. The quality control concentration was tested at low, medium and high levels. To measure the recovery rate, DAP metabolites were added to empty urine. Figure 2 shows the results of the analyte recoveries. The lyophilization procedure detection had a recovery of 40–90% for all analytes. QuEChERS had a recovery of 30–70%, while the best results were obtained with LLE, which had a recovery ranging from 93.18% to 101.98% for six DAP metabolites. Based on the recovery study, the LLE was selected for the further metabolites analysis in urine.

#### 2.2.5. Precision and Accuracy

To test the analytical procedure, we used three quality control samples spiked with DAP metabolites to measure precision and accuracy within a day (with different runs on the same day) and between days (continuous run on three consecutive days). Precision was reported as percentage RSD and accuracy as percentage recovery. The results showed that the values for precision were acceptable and ranged from 0.62 to 5.46% within a day and from 0.80 to 11.33% between days. Furthermore, the accuracy for the quality control samples ranged from 87.47% to 100.98% within a day and from 86.04% to 97.82% between days. Table 2 shows the precision and accuracy between and within days.

#### 2.2.6. Matrix Effect

**Figure 3:**
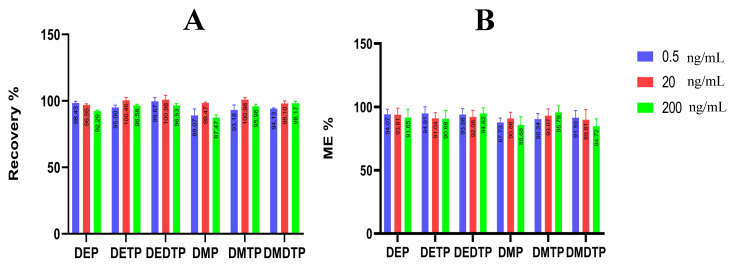
(**A**) Recovery and (**B**) matrix effect of DAP metabolites in three concentration ranges.

To evaluate the matrix effect, we compared the reaction of the analyte in the sample with a pure solvent. This comparison allowed us to determine the degree of ion suppression or the amplification factor of recovery, which we expressed as a percentage. The results, shown in Figure 3, indicate that the matrix effect was less than 20%. This percentage is within the acceptable range and indicates that only minimal ion suppression occurred.

#### 2.2.7. Stability

**Figure 4:**
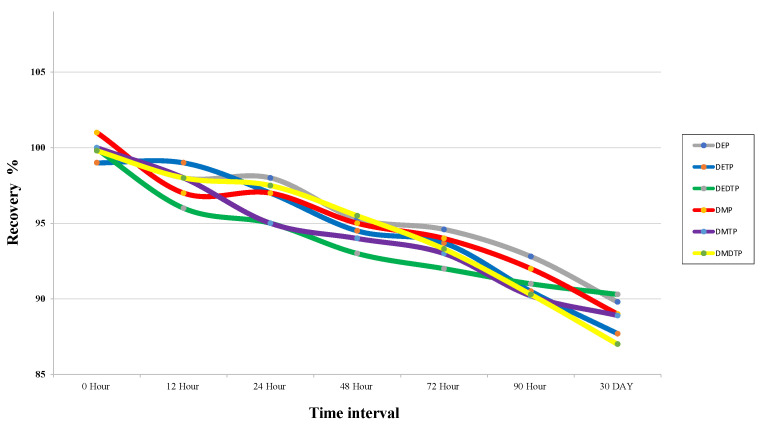
Stability assay of all target analytes at different time interval.

The stability of the analytes to temperature changes over time was tested. The changes were measured in the recovery rate and showed that less than 13% of the analytes reduced after 72 h at room temperature and 30 days at cold storage, as shown in Figure 4.

### 2.3. Method Application:

The study aim was to evaluate the efficacy of HFLC–MS/MS by analyzing 150 urine samples of adults from different villages in the Telangana region of India between 2021 and 2022. The liquid–liquid extraction procedure was used to confirm the reliability of the extraction process and the UFLC–MS/MS technique. Out of 150 samples, 100 were collected from individuals exposed to pesticides during cultivation, while 50 were collected from individuals residing in non-pesticide-exposed urban areas. During the study, DAP metabolites including DEP, DETP, DEDTP, DMP, DMTP and DMDTP were detected in the urine samples as shown in Table 3. The chromatogram of exposed urine samples has shown in Figure S5 (Supplementary).

After comparing DAP metabolites in two groups, the study found that individuals exposed to pesticides had higher concentrations of DAP metabolites during spraying. The total concentration of DAP metabolites found in the exposed samples was 56.37 ng/mL and 43.7 ng/mL in the unexposed samples, which means that DAP was higher in the exposed samples than in the unexposed samples, with differences about twice as large. These results suggest that people who are occupationally exposed to pesticides are at higher risk of exposure than the general population. The study confirms the accuracy and sensitivity of the current method and shows that it can provide reliable results. The comparison of exposed and control samples have given in Tables S1 and S2 (Supplementary). 

### 2.4. Discussion

This study presents a new UFLC–MS/MS method for the simultaneous detection of six endogenous DAP metabolites of organophosphate pesticides, including DEP, DETP, DEDTPDMP, DMTP andDMDTP, in urine samples. For quantification of DAP metabolites was performed by optimizing mass spectrometry and liquid chromatographic conditions. Base on the intensity and separation the negative ionization mode for mass separation used the MRM method to find our analytes, select precursor ions and their corresponding transitions, select two to four transitions for each analyte, and also, the set different mass parameters of DP, EP, CE and CXP were adjusted to improve MRM selectivity and obtain the most abundant precursors and productions. Liquid chromatographic separation of all six target analytes was performed by testing different columns (water, xstream C18- 3.5 µm, 4.6 mm × 150 mm, xstream C18- 150 × 4.6 mm, 4.5µm, Agilent C18 column- 3.5 mm × 50 mm × 3.5 µm) and mobile phase compositions with or without formic acid and buffers such as 10 mM ammonium acetate or 10 mM ammonium formate, and by elution programming with a wider range of temperature, flow rate and temperature combinations. After several rounds of optimization, a 6 min gradient elution was selected using an Agilent C18 column (2.1 mm × 50 mm × 1.7 µm), 10 mM ammonium formate and acetonitrile as the mobile phase, a column temperature of 40 °C and a flow rate of 0.5 mL/min, which separates all analytes and provides sharp chromatograms, accurate results and longer column life. With this method, there is a 5 min incubation period after each run to ensure that the column is thoroughly cleaned for the next run and to reduce carryover, which facilitates sample analysis.

In this study, three different sample preparation methods (lyophilization, QuEChERS and LLE) were compared to detect six DAP organophosphate metabolites. The recoveries of each method were evaluated against three quality control standards (low, medium and high). In the first method, a 10 mL urine sample was freeze-dried to remove water. The dried sample was then reconstituted with acetonitrile, dried again and filtered for analysis. The recoveries for this method ranged from 48.28% to 75.22%. In the second method, QuEChERS, a 10 mL urine sample was mixed with Na-acetate and MgSO4, shaken and centrifuged, and the resulting supernatant was then processed. The recoveries for this method ranged from 25.86% to 45.21%. The final method, liquid–liquid extraction, required only 200 µL of urine and used ethyl acetate. The recoveries ranged from 93% to 112%. Based on factors such as sample volume, solvent consumption, simplicity and recovery rate, it was determined that the present liquid–liquid extraction method was the preferred method for the analysis of DAP metabolites in urine. With this method it was possible to detect all six metabolites quickly and easily.

The detection of DAP metabolites was made possible by a rapid and straightforward liquid–liquid extraction procedure. In contrast to previous studies, which required 0.5–10 mL of sample and 5–15 mL of solvent for sample extraction, the recovery rate was lower (13–114%) and chromatographic separation was lengthy (10–35 min), with a detection limit of 0.1–6 ng/mL [17,33,37,40,41,42,43,44,45,46]. With the present method, each sample can be analyzed in only 30 min, making it ideal for laboratories that process a large volume of samples in a short time. In addition, this method is inexpensive and easy to use, making it a practical choice for any laboratory. Table 4 shows acomparison of the proposed method with other reported methods. The newly developed method had higher sensitivity, with detection limits for all analytes ranging from 0.0201 ng/mL to 0.0697 ng/mL. Recoveries ranged from 93% to 102%, indicating a more efficient extraction process than previous methods. The method exhibited excellent linearity, with a correlation coefficient (R^2^) of greater than 0.9923 over the entire calibration range of 0.1–200 ng/mL. The precision was also excellent, with relative standard deviations (RSD) below 15%. In addition, only 2 mL of solvent was required for sample preparation, whereas previous methods required more than 10 mL of different solvents, making the method less complicated and more environmentally friendly. Moreover, chromatographic separation was completed in the developed method in only 6 min, while other methods required a run time of 10–30 min.

The aim of this study was to compare the levels of DAP metabolites between two groups in India. The first group consisted of agricultural workers who are regularly exposed to pesticides, while the second group consisted of urban and rural residents who are not exposed to pesticides. The results showed that the concentrations of DEP, DMP, DMTP and DMDTP were higher in the exposed group, indicating that pesticide exposure has a significant impact on these metabolites. However, the concentrations of DETP and DEDTP were similar in both groups. Previous studies in India have mainly focused on the detection of metabolites in the urine DAP of men with abnormal semen [47], and children [39], but none have specifically studied farmers using organophosphate pesticides. This research method could help to assess the health effects of chronic pesticide exposure in farm workers and highlight the potential risks associated with their occupation. The study also signifies the importance of understanding the health effects of pesticides in order to develop appropriate interventions and protect the well-being of these workers.

## 3. Methods and Materials

### 3.1. Chemicals

Honeywell and JT Baker provided LC–MS grade water and acetonitrile (ACN). DAP metabolite standards include diethyl phosphate (DEP), diethyl thiophosphate (DETP) and diethyl dithiophosphate (DEDTP), dimethyl phosphate (DMP), dimethyl thiophosphate (DMTP) and dimethyl dithiophosphate (DMDTP), which were provided by the CDC, Atlanta, USA. Ammonium formate, ethyl acetate and formic acid were procured from Sigma-Aldrich India. Agilent Technology supplied an HPLC column, 0.2-micron syringe filters and 2 mLHPLC vials.

### 3.2. Preparation of Standards and Calibration Standard

To measure the DAP metabolites, a 1 ppm stock solution was prepared containing all six analytes in equal strength and dissolved in ACN. The solution was stored at −20 °C for later use. The stock solutions were serially diluted to obtain analyte concentrations in the range of 0.1–200 ng/mLto ensure accurate measurements and calibrations, with various calibration concentrations prepared including 0.1, 0.5, 1, 5, 10, 25, 50, 100, 150 and 200 ng/mL.

### 3.3. Sample Collection and Preparation

This study was approved by the Ethics Committee of ICMR-NIN, under the Ministry of Health and Family Welfare, Government of India. Participants gave their written informed consent after being informed about the study. The urine samples were collected in sterile plastic bottles with a volume of 50 mLon the first morning and then stored at −20 °C for later analysis. Three different extraction procedures (QuEChERS, lyophilization and LLE) were tested for the DAP metabolites. The best extraction procedure was selected for further investigation.

### 3.4. UFLC–MS/MS Conditions

The AB Sciex 4000 QTRAP and Shimadzu UFLC system with analysis software were used to detect DAP metabolites. Positive and negative electrospray ionization (ESI) conditions were tested for all analytes to determine the most effective ionization mode for each sample. The results showed that negative ionization gave the highest intensity, so negative ionization was chosen for all analytes. Specific mass parameters were chosen to fine-tune the multiple reaction monitoring (MRM) processes, including decluster potential (DP), entrance potential (EP), collision energy (CE) and cell exit potential (CXP), mass parameters such as the interfacial heating of 450 °C and ion spray voltage (IS) of −4500 eV. In addition, the curtain gas was set at 35 psi, while GS1 and GS2 were set at 45 and 40, respectively. The analytes were separated using an Agilent ZORBAX Stable Bond C18 reversed phase column with a size of 4.6 × 150 mm and a pore size of 1.8 µm. Different mobile phase compositions were tested, choosing 10 mM ammonium formate in water (solvent A) and ACN (solvent B). The binary pumps were configured for a flow rate of 500 µL/min and a sample injection volume of 20 µL was used. The gradient scheme used was as follows: 0.0–0.1 min at 90% B, 2.0–4.0 min at 100% B, 5–6 min at 90% B and 6–8 min at 5% A. The column was maintained at a constant temperature of 40 °C. Table 5 shows the mass parameters for all six DAP metabolites.

### 3.5. Method Validation

UFLC–MS/MS was developed following the method development approach described in the “Guidance Document on Pesticide Analytical Methods for Risk Assessment and Post-approval Control and Monitoring Purposes for Analytical Methods SANTE guidelines” [48]. The method was carefully crafted and several key parameters were considered in its development. These critical parameters include selectivity, linearity, recovery, carryover, calibration curve, lower limit of detection, lower limit of quantification, precision, accuracy, matrix effect and stability.

#### 3.5.1. Selectivity

The selectivity of the method was evaluated to investigate how different matrices and target substances affect it. Chromatograms of empty urine samples were compared with those of the target compounds to ensure accurate measurement and differentiation of the compounds of interest. Multiple reaction monitoring (MRM) in negative ionization mode was used to improve selectivity, allowing the precise monitoring of both parent and daughter fragments, as well as tailored techniques within the UFLC–MS/MS system.

#### 3.5.2. Linearity

In order to confirm that the analytical method is suitable for the intended use, a clear relationship must be established between the concentration of the substance to be analyzed and the results obtained in all applications. To evaluate linearity, the signals were plotted against the concentration changes of the analyzed substance. Standard calibration samples were prepared in batches with concentrations between 0.1 and 200 ng/mL. The peak area ratio of the analyzed substance (y) was plotted against the corresponding concentrations (x) using a weighted least squares linear regression (1/x).

#### 3.5.3. Recovery

In this study, the effectiveness of the sample extraction procedure was evaluated through a recovery study. During the recovery procedure, urine samples were spiked with three quality control concentrations (low, medium and high-0.5, 20 and 200 ng/mL). Each spiking consisted of five replicates and a range of standard mixtures of DAP metabolites were used. The percentage recovery for each spiked concentration was calculated using a previously described formula [49].

#### 3.5.4. LowerLimit of Detection and Quantification

The lower limit of detection (*LOD*) and the lower limit of quantification (*LOQ*) refer to the lowest concentration of the analyte that can be accurately measured with a signal-to-noise ratio. These limits are calculated by comparing the signals from samples with known low analyte concentrations with blank samples. A signal-to-noise ratio of 3:3 is commonly used to determine *LOD*, while a ratio of at least 10:1 is recommended for *LOQ* [49]. The formulas for *LOD* and *LOQ* are

LOD=3.3 x σ / S

LOQ=10 x σ / S

where *σ* is the standard deviation of the response and *S* is the slope of the calibration curve.

#### 3.5.5. Precision and Accuracy

To evaluate the accuracy and precision of the analytical method, three sets of quality control standards were tested. These sets contained different concentrations, ranging from low to high, within the same batch (intra-batch) and across three different batches per day for three days (inter-batch). The amount of each QC sample in each sample can be determined from calibration curves from the same batch. To measure precision, the RSD% between different batches can be used. Precision can also be determined by calculating the mean percentage recovery of a known amount of the analyte in a sample, the difference between the mean and the true value or the confidence interval. In addition, the precision of a method can be estimated by calculating the percentage RSD of repeated measurements on spiked urine samples.

#### 3.5.6. Matrix Effects

The matrix effect is critical to developing analytical methods, especially in techniques such as chromatography and mass spectrometry. It refers to the influence of the sample matrix on the analyte’s response, which can either enhance or suppress the analyte signal during the measurement process. This interference from the sample matrix can affect the accuracy and reliability of the quantification of the analytes. A known analyte concentration measured the matrix effect, and a standard matrix in equal amounts was added to the blank urine sample and measured using UFLC–MS/MS. The percentage recovery of the extraction was determined. The matrix effect (*ME*) was calculated as a percentage using this formula:

$$\%\;\mathrm{ME}\;=100-\left[\left(\mathrm{area}\;\mathrm{of}\;\mathrm{blank}\;\mathrm{matrix}/\mathrm{area}\;\mathrm{of}\;\mathrm{standard}\;\mathrm{solution}\right)\times 100\right]$$

#### 3.5.7. Stability

To test the stability of the samples, they were prepared at a constant concentration and tested for their temperature stability over short and long periods of time. To confirm their stability, the samples were kept in the autosampler for different periods of time: 0, 12, 24, 48, 72 and 90 h and 30 days at −20 °C.

## 4. Conclusions

A new UFLC–MS/MS method for measuring organophosphate pesticide metabolites in urine has been developed. The method is characterized by its sensitivity, selectivity, robustness and reproducibility. It is now possible to monitor human exposure to these pesticides more accurately. In India, this is the first method that can quantify all six DAP metabolites of OP pesticides in one run, enabling a comprehensive assessment of exposure. The developed method required a small sample volume of only 200 µL and liquid–liquid extraction; therefore, it can be used in any laboratory. The simplicity of the method and the increased sensitivity allow for the accurate measurement of biomarkers in urine that can be used in health impact assessments and provide a deeper understanding of the impact of these pesticides on human health.

## Figures and Tables

**Table 1:** Limits of detection (LODs), limits of quantitation (LOQs) and linearity for all target analytes.

Metabolites	Calibration Range (ng/mL)	R^2^	SLOP	LOD ng/mL	LOQ ng/mL
DEP	0.1–200	0.9997	9729.73	0.0201	0.0609
DETP	0.1–200	0.9992	8704.87	0.0323	0.0969
DEDTP	0.1–200	0.9964	19,603.50	0.0697	0.2112
DMP	0.1–200	0.9997	566.00	0.0207	0.0626
DMTP	0.1–200	0.9983	2196.01	0.0488	0.1479
DMDTP	0.1–200	0.9988	1365.28	0.0406	0.1229

The table shows the calibration range and R^2^ coefficient of covariance, LOD; limit of detection, LOQ; limit of quantitation.

**Table 2:** Precision and accuracy for all target analytes at three concentrations.

Metabolites	Concentration Added (ng/mL)	Inter-Day (*n* = 5)		Intra-Day (*n* = 15)	
		Accuracy (%) ± SD	Precision (RSD %)	Accuracy (%) ± SD	Precision (RSD%)
DEP	0.5	95.00 ± 1.87	1.97	86.04 ± 7.57	8.80%
	25	100.46 ± 2.09	2.08	97.60 ± 3.23	3.31%
	200	96.58 ± 0.86	0.89%	95.41 ± 1.34	1.40%
DETP	0.5	94.13 ± 0.59	0.62	89.76 ± 9.27	10.33
	25	98.10 ± 1.78	1.82	96.00 ± 2.49	2.60
	200	98.17 ± 1.46	1.48	96.99 ± 1.73	1.79
DMDTP	0.5	93.18 ± 3.73	4.00	90.51 ± 5.75	6.35
	25	100.98 ± 1.55	1.54	97.56 ± 3.53	3.62
	200	95.96 ± 1.52	1.58	96.85 ± 1.57	1.63
DMP	0.5	99.67 ± 2.84	2.85	89.76 ± 9.79	10.91
	25	100.98 ± 3.18	3.15	97.67 ± 4.25	4.35
	200	96.53 ± 1.48	1.53	94.60 ± 2.68	2.84
DMTP	0.5	98.43 ± 1.31	1.33	93.43 ± 10.60	11.33
	25	96.95 ± 0.80	0.82	96.95 ± 2.49	2.60
	200	92.29 ± 0.07	0.80	92.29 ± 0.74	0.80
DMDTP	0.5	89.07 ± 4.86	5.46	88.90 ± 8.07	9.07
	25	98.47 ± 0.62	0.63	97.82 ± 1.98	2.03
	200	87.47 ± 1.94	2.22	89.04 ± 2.21	2.48

*n* = number of observations, RSD relative standard deviation.

**Table 3:** Concentration of DAP metabolites in urine samples.

Metabolites	Concentration ng/mL Mean ± Sd Exposed	Concentration ng/mL Mean ± Sd Non-Exposed
DEP	13.04 ± 5.93	11.69 ± 4.97
DETP	1.25 ± 2.24	1.29 ± 1.51
DEDTP	0.58 ± 0.43	0.74 ± 0.62
DMP	16.88 ± 54.33	8.95 ± 4.41
DMTP	20.83 ± 21.60	18.19 ± 12.82
DMDTP	4.37 ± 7.27	2.84 ± 2.48
Total DAP	56.37 ± 59.69	43.71 ± 15.14

Table presents the mean and standard deviation (SD) of DAP metabolite concentrations among participants who were exposed to pesticides (*n* = 100) and those who were not exposed (*n* = 50).

**Table 4:** Comparative study of sample preparation and analytical method parameters.

Study	Extraction Method	Run Time (Minutes)	Solvent Volume (mL)	Sample Volume (mL)	Recovery (%)	LOD ng/mL
[40]	Liquid–liquid extraction	22	11	5	13–99	0.2
[37]	Solid-phase extraction	10	5	0.600	40–98	0.04–1.5
[41]	Liquid–liquid extraction	28	5	3	81–122	1–6
[42]	Liquid–liquid extraction (lyophilization)	35	10	10	99–100	0.02–0.09
[43]	Solid-phase extraction	10	15	1	80–100	0.1–0.4
[44]	Liquid–liquid extraction	15		1	54–101	0.03–1.77
[33]	Liquid–liquid extraction	20	8	4	70–112	0.50
[45]	Liquid–liquid microextraction	15	8	2	85.0–114	0.01–0.03
[46]	Solid-phase extraction	10	-	0.200	73–127	0.03–1.129
This study	Liquid–liquid extraction	6	2	0.200	93–102	0.02–0.06

The table compares various LC–MS/MS methods used for analyzing DAP metabolites. The methods are evaluated based on their extraction technique, analysis duration, solvent usage, detection limit and recovery percentage. Our research employed liquid–liquid extraction that took 6 min and necessitated 2 mL of solvent. The limit of detection was 0.02–0.06 µg/mL, and the recovery percentage was 93–102%.

**Table 5:** MRM parameters for DAP metabolites.

Metabolites	Parent Ions (*m*/*z*)	Product Ions (*m*/*z*)	DP	CE	CXP	RT
DEP	152.9	78.9/125	−47	−26	−1.5	2.44
DETP	168.8	140.8/95	−54	−18	−9.7	1.97
DEDTP	184.7	110.8/157	−54	−29	−3.3	1.92
DMP	125	62.8/110	−68	−4	−2	2.38
DMTP	140.8	125.8/96	−61	−19	−1	1.96
DMDTP	156.7	112/142	−56	−23	−10	1.92

Table illustrates the transition from precursor to product ion and includes all mass parameters for the individual analyst DP, including decluster potential (DP), collision energy (CE), cell exit potential (CXP) and retention time (RT).

## Data Availability

The data are made available on request. The majority of the data is stored in the Supplementary File.

## Supplementary Materials

The following supporting information can be downloaded at: https://www.mdpi.com/article/10.3390/molecules28155800/s1, Figure S1: The chemical structure of six DAP metabolites; Figure S2: The linearity diagram of all DAP metabolites; Figure S3: The carry-over study; Figure S4: Comparative extraction procedures (QuEChERS, lyophilization and liquid-liquid extraction); Figure S5: The chromatogram of the real urine sample analyzed for DAP metabolites; Table S1 Group statistics of the exposed sample (100) and the control (50); Table S2 Data of 100 exposed and 50 control samples with their six DAP metabolite concentrations.
