# Supplementary material for: Development and Validation of a New UFLC–MS/MS Method for the Detection of Organophosphate Pesticide Metabolites in Urine
Source: Molecules. 2023 Aug 1;28(15):5800. doi: 10.3390/molecules28155800 (PMC10421278; doi:10.3390/molecules28155800)
Supplement: Supplementary file 1 [file molecules-28-05800-s001.zip › molecules-2507668-supplementary.pdf]

Supplementary files

Figure S1: The chemical structure of six DAP metabolites

Figure S2: The linearity diagram of all DAP metabolites

Figure S3: The carry-over study

Figure S4: Comparative extraction procedures (QuEChERS, lyophilisation and liquid-liquid extraction)

Figure S5: The chromatogram of the real urine sample analysed for DAP metabolites

Table:1S. Group statistics of the exposed sample (100) and the control (50)

Table:S2 Data of 100 exposed and 50 control samples with their six DAP metabolite concentrations.

Figure S1: The chemical structure of six DAP metabolites

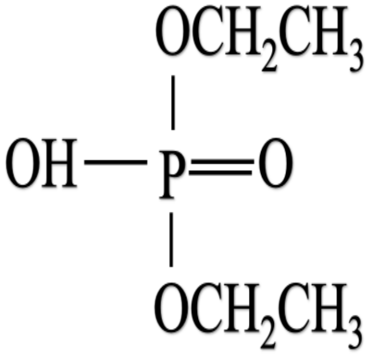

Diethyl phosphate (DEP)

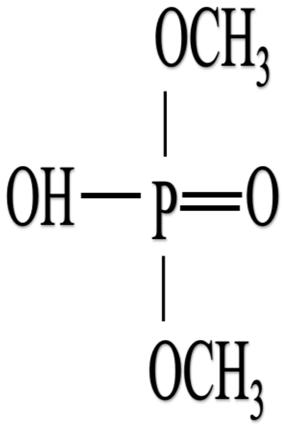

Dimethyl phosphate (DMP)

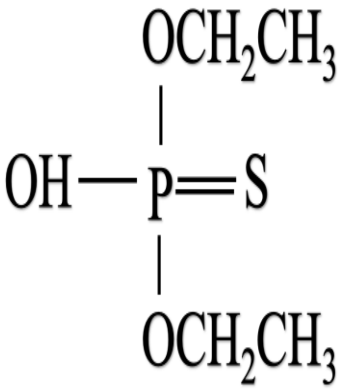

Diethyl thiophosphate (DETP)

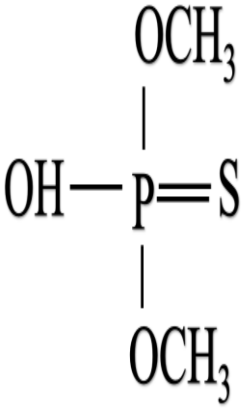

Dimethyl thiophosphate (DMTP)

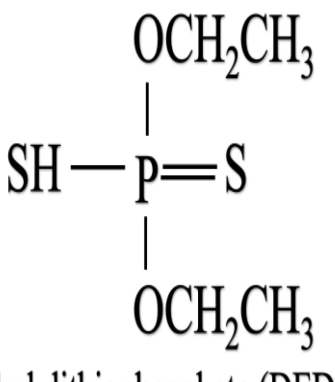

Diethyl dithiophosphate (DEDTP)

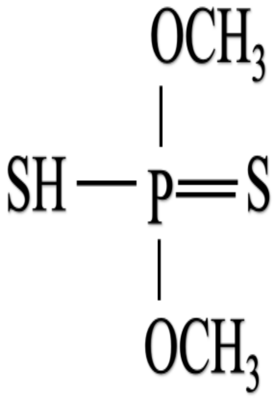

Dimethyl dithiophosphate (DMDTP)

Figure S2: The linearity diagram of all DAP metabolites

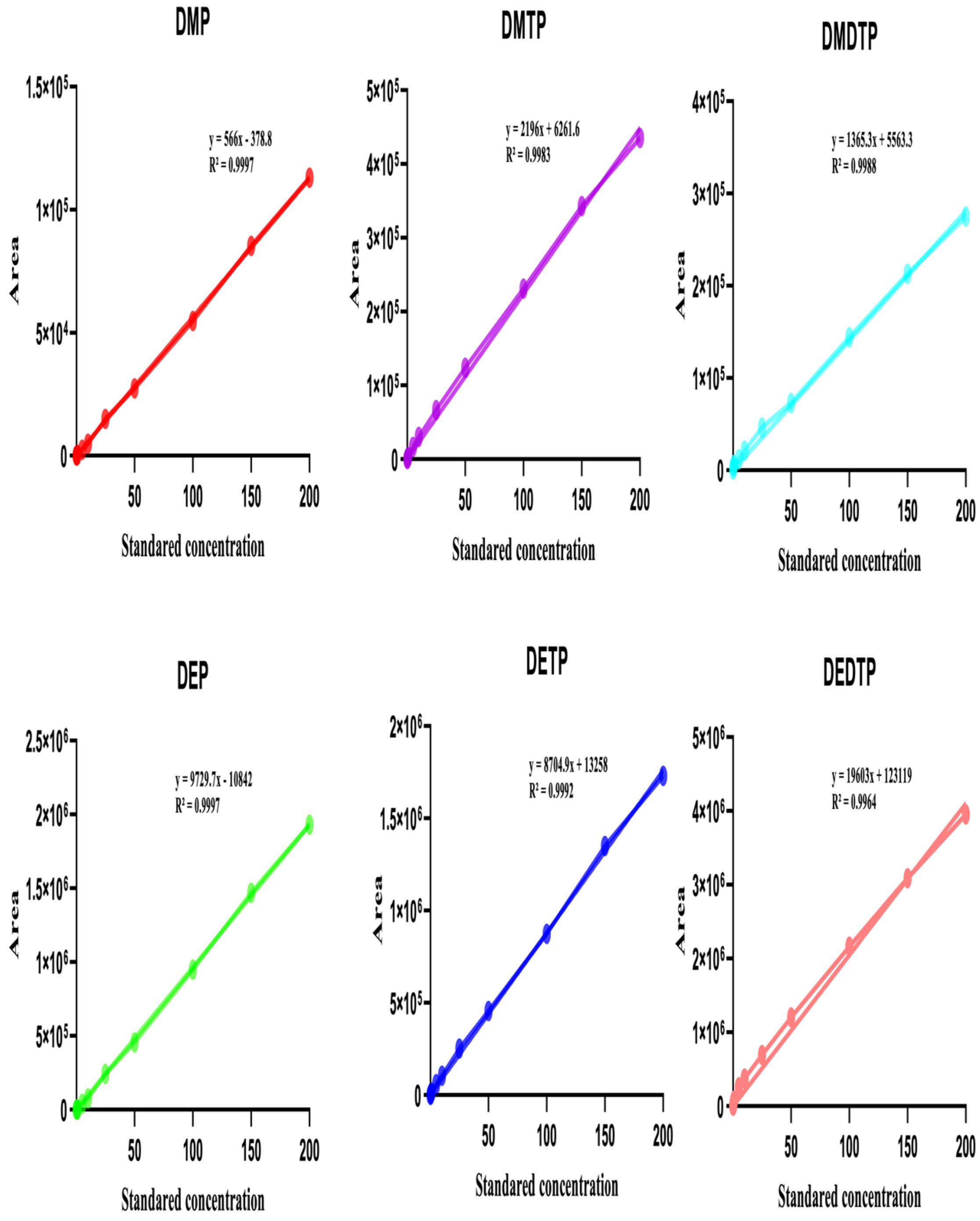

Figure S3: The carry-over study

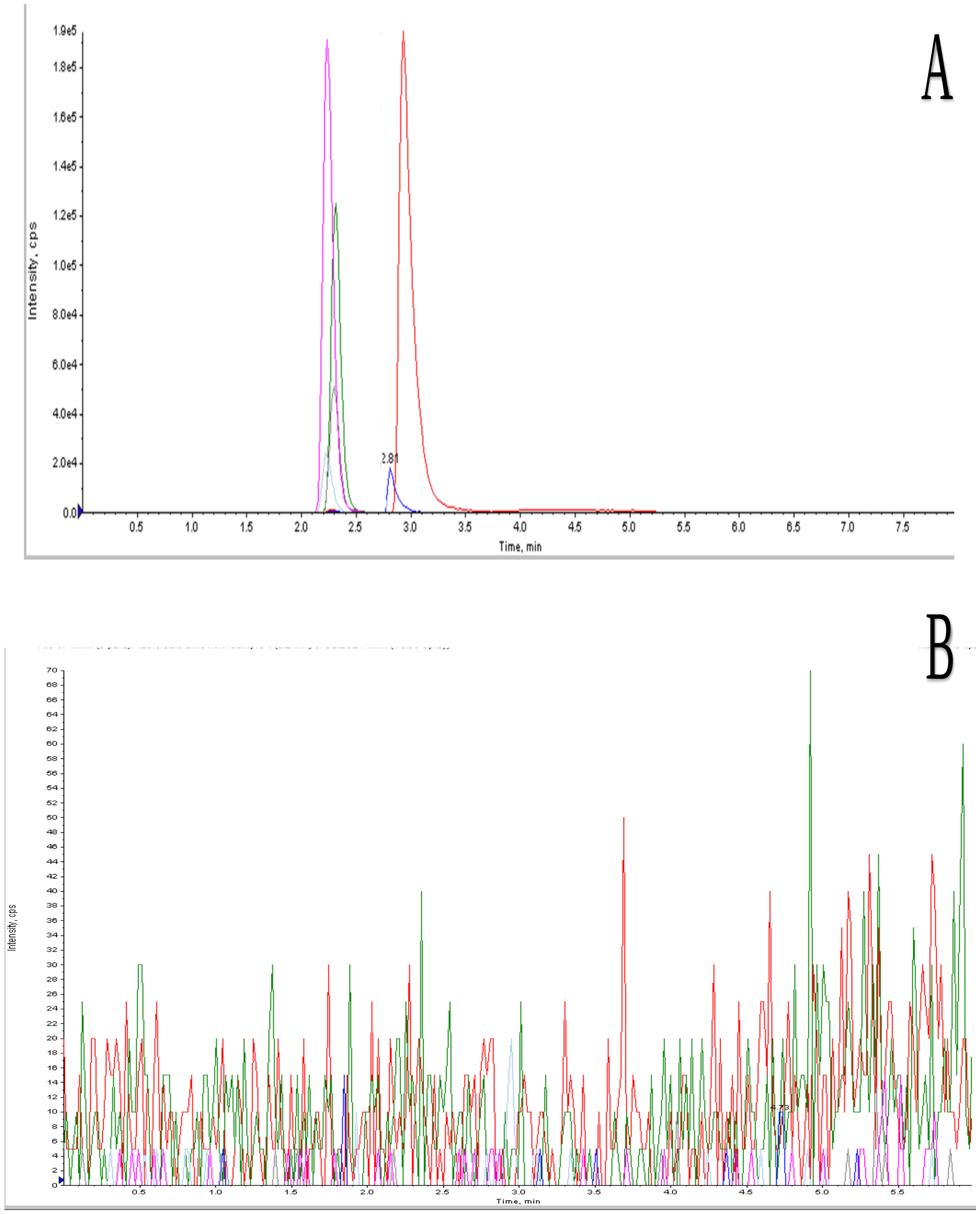

Based on the carryover study, which involved the analysis of a 200 ng/mL standard sample, it was observed that after B examined the blank (mobile phase), there were no other interfering analytes carried forward to the subsequent chromatographic run.

Figure S4: Comparative extraction procedures (QuEChERS, lyophilisation and liquid-liquid extraction)

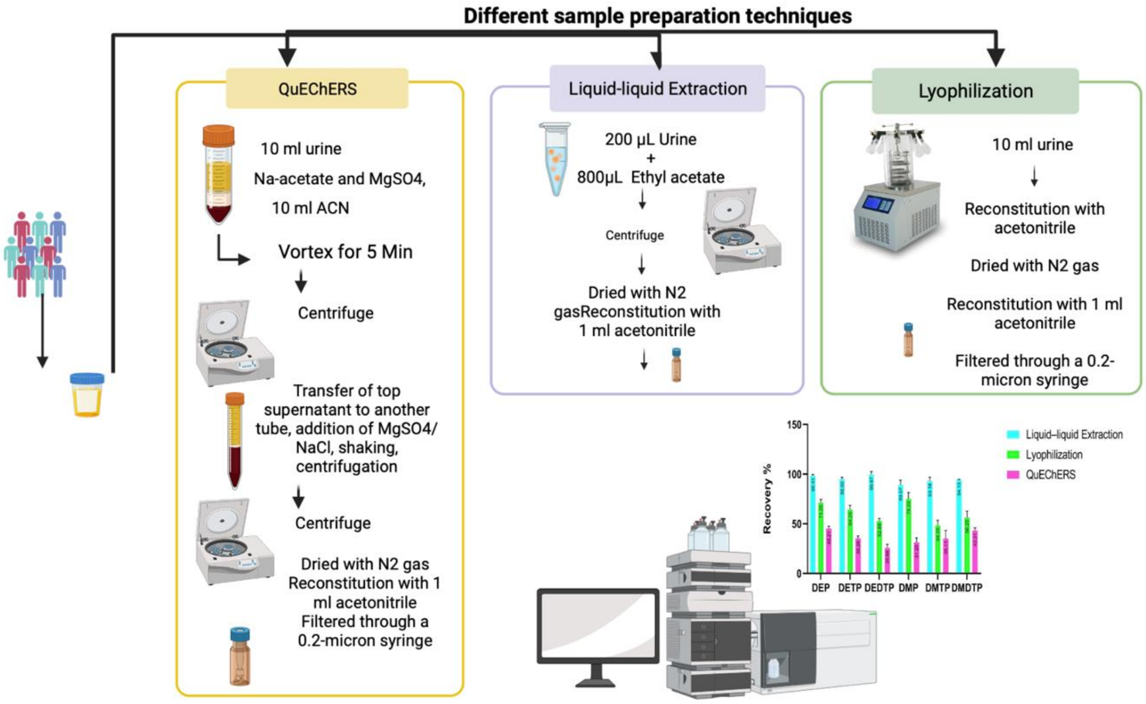

For each extraction process in the lyophilization method, a series of procedures were carried out. The dried sample was then combined with different solvents such as H2O, methanol, and a mixture of H2O and ACN (acetonitrile). The best solvent for extraction was chosen for the subsequent recovery study. In the official AOAC QuEChERS (Quick, Easy, Cheap, Effective, Rugged, and Safe) method, the prescribed procedures were followed strictly to ensure accurate and reliable results. Various solvents including hexane, ethyl ether, methanol, and benzene were used for liquid-liquid extraction (LLE). Ethyl acetate was found to be the most favourable solvent for extraction after comparative analysis.

Figure S5: The chromatogram of the real urine sample analysed for DAP metabolites

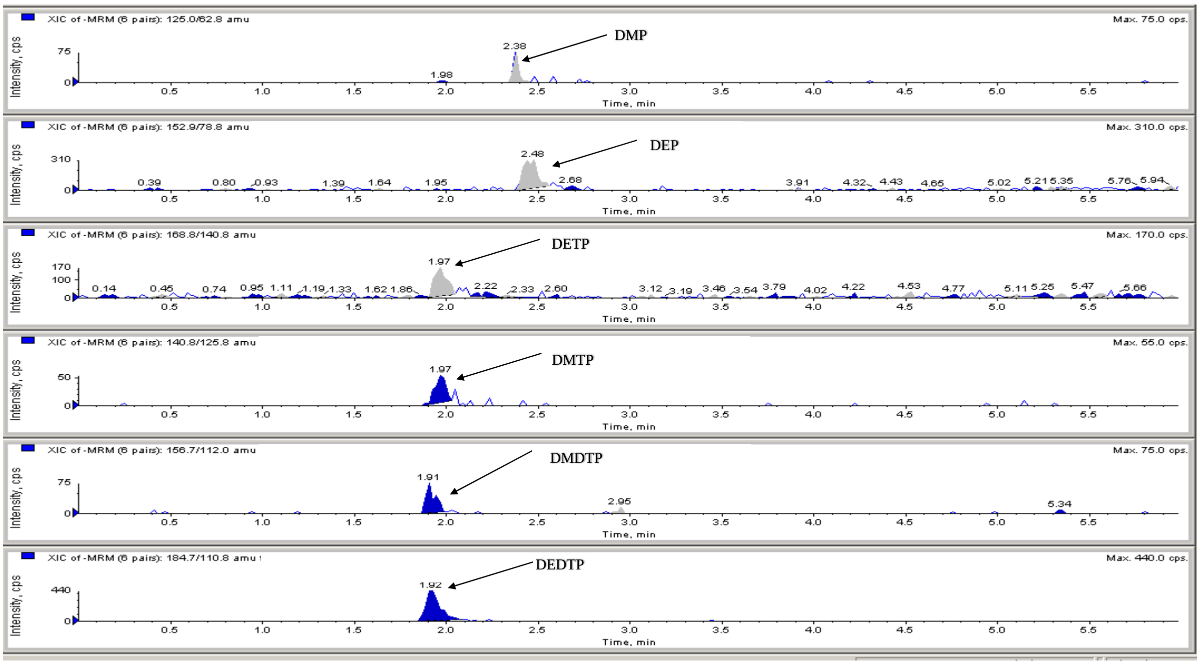

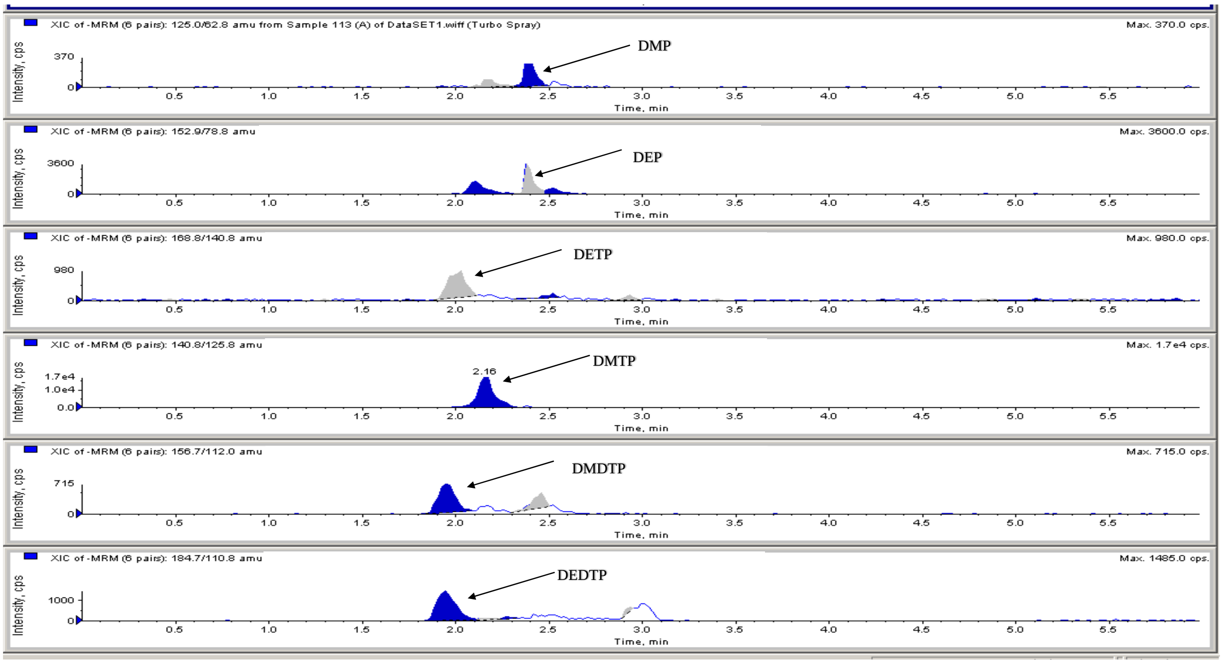

Table:1S. Group statistics of the exposed sample (100) and the control (50)

**Group Statistics**					
SAMPLE TYPE		N	Mean	Std. Deviation	Std. Error Mean
DEP	exposed	100	13.03515	5.932902	0.593290
	control	50	11.69200	4.972661	0.703240
DETP	exposed	100	1.247381	2.2372394	0.2237239
	control	50	1.288408	1.5132391	0.2140043
DEDTP	exposed	100	0.58306	0.430734	0.043073
	control	50	0.73642	0.622833	0.088082
DMP	exposed	100	16.8787	54.32874	5.43287
	control	50	8.9518	4.40802	0.62339
DMTP	exposed	100	20.833630	21.6048938	2.1604894
	control	50	18.194852	12.8176958	1.8126959
DMDTP	exposed	100	4.37326	7.265589	0.726559
	control	50	2.84300	2.484496	0.351361

Independent Samples Test											
		Levene's Test for Equality of Variances		t-test for Equality of Means							
		F	Sig.	t	df	Significance		Mean Difference	Std. Error Difference	95% Confidence Interval of the Difference	
						One-Sided p	Two-Sided p			Lower	Upper
DEP	Equal variances assumed	1.033	0.311	1.377	148	0.085	0.171	1.343150	0.975688	-0.584930	3.271230
	Equal variances not assumed			1.460	114.792	0.074	0.147	1.343150	0.920076	-0.479379	3.165679
DETP	Equal variances assumed	0.039	0.845	-0.117	148	0.454	0.907	-0.0410270	0.3509803	-0.7346071	0.6525531
	Equal variances not assumed			-0.133	134.888	0.447	0.895	-0.0410270	0.3095969	-0.6533190	0.5712650
DEDTP	Equal variances assumed	4.409	0.037	-1.762	148	0.040	0.080	-0.153360	0.087041	-0.325364	0.018644
	Equal variances not assumed			-1.564	73.166	0.061	0.122	-0.153360	0.098050	-0.348765	0.042045

DMP	Equal variances assumed	2.959	0.087	1.028	148	0.153	0.305	7.92690	7.70874	-7.30651	23.16031
	Equal variances not assumed			1.450	101.588	0.075	0.150	7.92690	5.46852	-2.92042	18.77422
DMTP	Equal variances assumed	2.417	0.122	0.796	148	0.214	0.428	2.6387780	3.3164438	-3.9149213	9.1924773
	Equal variances not assumed			0.936	143.634	0.176	0.351	2.6387780	2.8202094	-2.9356979	8.2132539
DMDTP	Equal variances assumed	7.178	0.008	1.446	148	0.075	0.150	1.530260	1.058609	-0.561681	3.622201
	Equal variances not assumed			1.896	135.722	0.030	0.060	1.530260	0.807058	-0.065775	3.126295

Table:S2 Data of 100 exposed and 50 control samples with their six DAP metabolite concentrations.

SAMPLE CODE	Age	Sex	EDUCATION	Tobacco	ALCOHOL	DMP ng/mL	DEP ng/mL	DETP ng/mL	DMTP ng/mL	DMDTP ng/mL	DEDTP ng/mL
**EXP 1**	50	Male	Illiterate	No	Yes	2.81	10.700	1.4600	20.3000	2.120	0.629
**EXP 2**	34	Female	5-10 std	No	Yes	4.96	6.540	0.3440	3.3200	2.050	0.342
**EXP 3**	37	Male	Illiterate	Yes	Occasionally	3.98	18.400	1.5600	25.1000	2.140	0.785
**EXP 4**	48	Male	1-4 std	No	Yes	4.62	19.200	0.3870	20.3000	2.120	0.405
**EXP 5**	29	Male	College	No	No	3.47	7.890	0.2350	11.1000	2.120	0.362
**EXP 6**	74	Male	5-10 std	No	Yes	8.75	8.150	0.1660	6.9400	2.100	0.415
**EXP 7**	32	Female	1-4 std	No	Yes	2.87	11.400	0.9240	25.5000	2.070	0.529
**EXP 8**	45	Female	Illiterate	No	Toddy	4.28	9.830	1.3900	0.2330	2.090	0.404
**EXP 9**	35	Female	Illiterate	No	No	3.63	15.200	0.4930	11.6000	2.110	0.421
**EXP 10**	48	Female	Illiterate	Yes	No	4.89	15.300	0.9520	6.5400	2.050	0.429
**EXP 11**	54	Female	Illiterate	No	Yes	2.58	2.770	0.0670	7.7200	2.110	0.363
**EXP 12**	58	Male	5-10 std	No	Yes	7.38	15.700	1.2700	15.8000	1.093	0.576
**EXP 13**	44	Female	Illiterate	No	Yes	7.99	14.600	0.5280	11.0000	2.150	0.412
**EXP 14**	46	Male	5-10 std	No	Yes	5.54	10.300	3.3100	11.9000	2.030	0.890
**EXP 15**	42	Male	College	No	No	20.80	12.200	1.1300	11.2300	2.120	0.676
**EXP 16**	35	Female	5-10 std	No	Yes	6.75	12.900	0.4930	22.4000	2.080	0.388
**EXP 17**	70	Male	Illiterate	No	No	2.67	20.400	0.3480	15.5000	15.500	0.345
**EXP 18**	49	Male	5-10 std	No	Yes	346.00	37.500	0.4490	10.8000	10.800	0.481

**EXP 19**	40	Male	11-12 std	Yes	Yes	4.74	21.100	3.0560	6.4200	64.200	0.040
**EXP 20**	28	Female	5-10 std	Yes	Yes	4.26	16.000	0.3630	11.7000	11.700	0.406
**EXP 21**	38	Female	1-4 std	No	No	7.58	16.300	0.4150	20.0000	20.000	0.373
**EXP 22**	42	Male	11-12 std	No	Toddy	6.11	21.500	0.8160	11.9000	1.100	0.786
**EXP 23**	50	Male	5-10 std	No	No	1.92	6.680	0.2080	3.4600	3.460	0.421
**EXP 24**	52	Male	1-4 std	No	Yes	5.54	7.240	0.3500	9.5500	1.950	0.357
**EXP 25**	57	Female	Illiterate	No	No	7.16	7.480	0.3600	7.9500	7.950	0.412
**EXP 26**	35	Female	Illiterate	No	No	10.80	4.400	0.2720	6.5100	6.510	0.383
**EXP 27**	52	Male	1-4 std	No	Yes	2.26	11.750	0.0692	7.2600	7.260	0.367
**EXP 28**	32	Female	Illiterate	No	No	5.75	9.970	0.9800	22.3000	22.300	0.358
**EXP 29**	67	Male	5-10 std	No	No	9.09	6.750	0.3440	10.6000	10.600	0.386
**EXP 30**	51	Male	5-10 std	Yes	Yes	4.35	22.600	2.2600	16.9000	1.690	0.639
**EXP 31**	40	Male	5-10 std	No	Yes	16.90	15.700	0.6850	14.1000	14.100	0.445
**EXP 32**	38	Male	5-10 std	No	No	3.08	20.200	1.2900	28.2000	2.280	0.422
**EXP 33**	60	Male	Illiterate	No	Yes	1.40	13.300	0.9320	21.5000	2.110	0.373
**EXP 34**	40	Male	5-10 std	No	No	7.51	7.950	0.4890	8.5800	8.580	0.465
**EXP 35**	40	Male	1-4 std	No	Yes	7.11	10.700	0.2900	2.6800	2.680	0.394
**EXP 36**	35	Male	College	No	No	3.82	17.300	0.2050	11.5000	11.500	0.380
**EXP 37**	23	Male	College	No	Yes	434.00	11.000	0.9100	4.9500	4.950	0.564
**EXP 38**	57	Male	Illiterate	Yes	yes	6.25	13.900	1.2600	5.9600	5.600	0.846
**EXP 39**	25	Male	College	No	Yes	22.40	4.520	0.4320	14.6000	13.600	0.372

**EXP 40**	36	Male	College	No	Yes	10.60	17.200	0.3010	7.0000	7.030	0.665
**EXP 41**	29	Male	11-12 std	No	Yes	28.50	13.200	0.6000	31.1000	2.140	0.518
**EXP 42**	54	Male	Illiterate	Yes	Occasionally	8.66	6.410	0.2500	11.8000	2.120	0.392
**EXP 43**	40	Male	5-10 std	No	Yes	12.98	14.000	0.3470	12.4000	2.080	0.369
**EXP 44**	30	Male	11-12 std	No	Yes	8.10	16.990	0.1770	19.1000	2.150	0.388
**EXP 45**	60	Male	Illiterate	Yes	Toddy	6.25	9.770	1.0500	5.8600	2.140	0.579
**EXP 46**	50	Male	1-4 std	No	Occasionally	10.30	8.150	0.2930	16.9000	2.120	0.400
**EXP 47**	48	Male	Illiterate	No	Yes	9.51	9.840	0.0741	2.9600	2.160	0.425
**EXP 48**	36	Male	11-12 std	Yes	Occasionally	7.67	10.400	1.0700	44.4000	2.150	0.770
**EXP 49**	40	Male	5-10 std	No	Toddy	3.34	10.200	0.6910	10.1000	2.080	0.395
**EXP 50**	65	Male	Illiterate	No	Yes	3.08	16.200	0.3240	13.2000	2.150	0.359
**EXP 51**	70	Male	Illiterate	No	Toddy	8.45	13.400	0.2510	3.7700	2.080	0.412
**EXP 52**	62	Male	Illiterate	Yes	Occasionally	11.10	8.820	0.5330	7.7000	2.050	0.445
**EXP 53**	30	Male	5-10 std	Yes	Occasionally	9.66	11.800	0.8220	6.9100	2.130	0.383
**EXP 54**	55	Male	Illiterate	No	Yes	6.24	5.040	0.9830	12.7000	2.100	0.351
**EXP 55**	37	Male	5-10 std	No	Yes	7.41	5.570	1.0930	30.4000	2.110	0.386
**EXP 56**	27	Male	Illiterate	No	Yes	14.90	21.000	1.2600	26.4000	2.100	0.749
**EXP 57**	55	Male	Illiterate	No	Yes	6.24	15.750	3.8700	144.0000	2.180	0.660
**EXP 58**	70	Male	Illiterate	Yes	Yes	7.98	11.740	0.2820	17.4000	2.110	0.491
**EXP 59**	65	Male	Illiterate	Yes	Yes	7.33	11.900	0.7740	25.6000	2.110	0.672
**EXP 60**	54	Male	Illiterate	Yes	Yes	7.28	16.700	0.3690	29.3000	2.170	0.692

**EXP 61**	70	Male	1-4 std	Yes	Yes	6.93	15.300	0.1480	37.4000	2.110	0.399
**EXP 62**	60	Male	Illiterate	Yes	Yes	6.58	1.640	0.7750	13.3000	2.090	1.920
**EXP 63**	70	Male	Illiterate	No	Yes	10.50	12.100	1.0200	36.0000	2.110	0.750
**EXP 64**	70	Male	Illiterate	Yes	Yes, Toddy	13.00	14.700	0.3350	34.5000	2.070	0.377
**EXP 65**	35	Male	11-12 std	No	Yes	6.84	6.990	0.3880	12.8000	2.030	0.425
**EXP 66**	58	Male	Illiterate	No	Yes	10.85	8.750	0.4960	57.2000	2.170	0.384
**EXP 67**	39	Male	5-10 std	No	Occasionally	10.96	4.870	1.0280	37.3000	2.090	1.130
**EXP 68**	50	Male	5-10 std	No	Yes	8.28	15.000	1.2500	74.3000	2.140	0.437
**EXP 69**	28	Male	5-10 std	No	Occasionally	12.08	17.600	2.3200	103.0000	2.130	1.450
**EXP 70**	65	Male	College	Yes	Occasionally	14.10	8.580	0.8220	32.3000	2.070	0.457
**EXP 71**	65	Male	5-10 std	Yes	Stopped	17.45	8.720	2.7600	63.8000	2.150	1.790
**EXP 72**	60	Male	Illiterate	Yes	Yes	31.90	14.100	0.1190	8.1600	2.190	0.355
**EXP 73**	66	Male	11-12 std	No	No	6.37	9.300	0.2950	21.3000	2.130	0.439
**EXP 74**	50	Male	5-10 std	No	Yes	7.85	13.500	2.0400	55.0000	2.170	2.940
**EXP 75**	41	Male	5-10 std	No	Occasionally	9.29	6.450	0.9380	12.0000	2.110	0.756
**EXP 76**	52	Male	11-12 std	Yes	Occasionally	34.80	10.200	6.2900	73.4000	2.130	1.080
**EXP 77**	50	Male	5-10 std	Yes	Toddy	11.94	10.900	1.5400	33.1000	2.140	0.980
**EXP 78**	45	Male	Illiterate	Yes	Yes	9.53	13.600	0.7520	10.1000	2.160	0.366
**EXP 79**	37	Male	Illiterate	Yes	Yes	16.50	15.800	0.0733	31.9000	2.160	0.349
**EXP 80**	40	Male	5-10 std	Yes	Occasionally	7.98	24.700	0.5900	29.1000	2.050	0.444
**EXP 81**	52	Male	5-10 std	No	No	9.19	10.500	2.4500	34.9000	12.190	2.580

**EXP 82**	42	Male	Illiterate	No	Occasionally	7.37	9.450	0.1370	56.0000	12.120	0.746
**EXP 83**	45	Male	College	No	Occasionally	7.19	11.200	6.7800	19.6000	2.070	1.650
**EXP 84**	65	Male	Illiterate	No	Yes	6.83	20.700	0.4680	44.0000	2.190	0.477
**EXP 85**	56	Male	5-10 std	Yes	everyday	9.93	30.500	1.0100	10.9000	2.140	0.508
**EXP 86**	45	Male	1-4 std	Yes	Yes	13.30	9.830	1.3000	12.9000	2.160	0.581
**EXP 87**	35	Female	1-4 std	No	No	29.60	11.200	0.6010	13.9000	2.150	0.561
**EXP 88**	30	Male	College	Yes	Occasionally	26.80	10.520	0.4090	20.0000	2.180	0.363
**EXP 89**	52	Male	5-10 std	Yes	everyday	29.30	22.050	2.9100	3.9500	2.130	0.466
**EXP 90**	32	Male	5-10 std	No	Occasionally	6.90	16.600	0.1935	8.5000	2.100	0.364
**EXP 91**	64	Male	5-10 std	No	No	6.15	3.045	0.4105	0.5950	2.150	0.370
**EXP 92**	50	Male	5-10 std	No	No	8.65	9.900	0.4010	14.8500	2.090	0.394
**EXP 93**	43	Male	1-4 std	Yes	Yes	7.40	9.550	0.7550	13.3000	2.120	0.401
**EXP 94**	52	Male	5-10 std	No	Occasionally	6.50	14.365	1.6550	1.6700	2.160	0.384
**EXP 95**	60	Male	Illiterate	No	No	7.45	13.755	0.4685	3.7350	2.110	0.462
**EXP 96**	80	Male	1-4 std	No	No	8.20	25.050	2.2300	6.7700	2.120	0.522
**EXP 97**	42	Male	5-10 std	Yes	Toddy	8.85	17.650	17.6000	47.0000	2.100	0.397
**EXP 98**	70	Male	Illiterate	No	Toddy	9.50	25.150	11.3000	9.2200	2.013	0.497
**EXP 99**	55	Male	Illiterate	Yes,	No	7.45	15.250	1.5200	19.8500	2.050	0.383
**EXP 100**	50	Female	Illiterate	No	No	6.00	15.050	1.2540	2.8600	2.150	0.430
**CONT 1**	36	Male			Yes	7.85	9.740	0.9260	22.5000	2.090	0.614
**CONT 2**	70	Male	1-4 std	Yes	Yes	13.32	10.200	0.7510	7.2000	2.140	0.449

**CONT 3**	45	Female	Illiterate	No	No	12.94	5.280	0.1460	4.2000	2.150	0.335
**CONT 4**	52	Male	College	Yes	Yes	3.35	4.010	6.7940	21.1000	2.160	0.649
**CONT 5**	54	Male	Illiterate	No	Yes	5.62	18.200	0.6850	33.1000	2.150	0.586
**CONT 6**	35	Female	Illiterate	No	No	4.95	10.720	0.2920	8.5500	2.150	0.356
**CONT 7**	45	Female	Illiterate	No	Yes	4.21	12.330	0.2300	4.3300	2.110	0.350
**CONT 8**	57	Male	5-10 std	Yes	Yes	2.54	20.700	0.0942	16.3000	2.070	0.395
**CONT 9**	27	Female	11-12 std	No	No	4.02	4.420	0.7020	5.2800	2.240	0.734
**CONT 10**	43	Female	5-10 std	No	Yes	4.89	16.200	0.3510	19.0000	2.190	0.374
**CONT 11**	50	Female	Illiterate	Yes	Occasionally	5.97	10.100	0.9550	40.0000	2.050	0.396
**CONT 12**	56	Male	5-10 std	No	Yes	5.89	13.400	0.7790	28.7000	2.030	0.665
**CONT 13**	60	Female	Illiterate	No	Yes	3.14	4.910	6.0682	1.5900	2.170	0.505
**CONT 14**	28	Female	5-10 std	No	No	11.74	5.830	0.3770	20.0000	2.130	0.525
**CONT 15**	45	Female	Illiterate	Yes	yes	12.44	9.120	0.6190	16.4000	16.400	1.500
**CONT 16**	55	Male	Illiterate	Yes	Toddy	6.54	3.570	0.1370	9.5700	9.570	0.326
**CONT 17**	45	Female	Illiterate	Yes	Yes	18.35	10.500	1.1200	9.6100	9.610	0.599
**CONT 18**	46	Male	5-10 std	Yes	Yes	4.91	12.200	0.4920	13.2000	2.530	0.357
**CONT 19**	28	Female	5-10 std	No	No	5.64	16.580	0.6660	19.9000	1.090	0.447
**CONT 20**	25	Male	College	No	Yes	13.43	16.100	0.7040	34.9000	4.140	0.678
**CONT 21**	33	Male	College	No	Yes	5.02	9.650	0.3040	10.7000	2.800	0.415
**CONT 22**	70	Male	College	Yes	Occasionally	14.60	10.200	0.6570	15.2000	3.150	0.520
**CONT 23**	60	Male	Illiterate	No	No	11.92	8.890	0.3310	18.9000	2.160	0.354

**CONT 24**	38	Male	Illiterate	No	No	5.23	23.940	1.7400	28.3000	3.360	0.671
**CONT 25**	48	Male	Illiterate	No	Yes	12.35	18.800	3.4900	43.4000	2.120	1.661
**CONT 26**	45	Male	5-10 std	No	No	13.52	15.000	0.6430	9.9400	2.050	0.497
**CONT 27**	65	Male	1-4 std	Yes	No	12.82	3.910	1.0900	21.6000	3.010	0.642
**CONT 28**	60	Male	Illiterate	Yes	Yes	5.19	10.950	0.8070	28.9000	2.110	0.587
**CONT 29**	55	Female	Illiterate	No	No	12.37	16.100	0.3170	15.5000	2.110	0.366
**CONT 30**	55	Female	Illiterate	No	No	4.69	12.600	0.3170	7.4100	2.140	2.413
**CONT 31**	60	Male	Illiterate	No	Yes	14.53	3.040	0.1350	12.7000	2.100	0.368
**CONT 32**	50	Female	Illiterate	No	No	4.01	13.700	5.6610	31.1000	2.100	0.437
**CONT 33**	45	Female	5-10 std	No	No	15.28	10.000	1.6200	48.2000	2.130	0.475
**CONT 34**	37	Male	5-10 std	No	No	4.80	9.920	2.1400	48.1000	2.090	0.431
**CONT 35**	52	Male	5-10 std	Yes	Yes	13.55	8.050	1.2900	0.2530	2.130	0.471
**CONT 36**	40	Male	5-10 std	No	Yes	13.43	8.080	1.4100	8.1500	2.110	0.348
**CONT 37**	30	Male	Illiterate	Yes	Yes	14.43	7.880	1.3080	22.4000	2.070	1.361
**CONT 38**	51	Male	11-12 std	Yes	Yes	6.46	13.800	0.4490	12.8000	2.040	0.468
**CONT 39**	39	Female	5-10 std	No	No	9.83	12.500	0.6240	0.0396	2.110	0.545
**CONT 40**	50	Male	Illiterate	No	Yes	13.68	12.200	2.5900	3.6400	4.080	2.530
**CONT 41**	65	Male	Illiterate	No	No	5.99	11.900	2.7090	37.5000	2.100	0.407
**CONT 42**	47	Male	5-10 std	Yes	Yes	12.05	11.900	1.3100	27.2000	2.130	0.551
**CONT 43**	39	Male	College	No	No	5.77	13.800	0.4450	10.6300	2.130	0.742
**CONT 44**	50	Male	Illiterate	Yes	Yes	12.18	15.040	0.5980	2.8000	2.130	1.586

**CONT 45**	35	Male	5-10 std	No	Yes	14.56	18.100	2.2780	25.3000	2.110	0.416
**CONT 46**	70	Male	Illiterate	Yes	Yes	5.61	20.900	0.8680	28.3000	2.090	0.578
**CONT 47**	60	Male	5-10 std	Yes	Yes	5.20	13.200	4.1000	1.4100	2.090	0.677
**CONT 48**	65	Male	Illiterate	No	Yes	5.93	4.140	0.2180	18.0000	2.120	1.362
**CONT 49**	48	Male	1-4 std	No	Yes	15.70	19.700	0.6930	3.8400	2.090	0.710
**CONT 50**	30	Male	College	Yes	Toddy	5.15	12.600	1.3900	32.1000	2.020	3.392

- EXP = exposed
- CONT = control
